# Frequency Management for Electromagnetic Continuous Wave Conductivity Meters

**DOI:** 10.3390/s16040490

**Published:** 2016-04-07

**Authors:** Przemyslaw Mazurek, Grzegorz Putynkowski

**Affiliations:** 1 Department of Signal Processing and Multimedia Engineering, West Pomeranian University of Technology Szczecin, 26. Kwietnia St., Szczecin 71126, Poland; 2 CBRTP SA, Zlota 59 St., Warsaw 00120, Poland; grzegorz.putynkowski@cbrtp.pl

**Keywords:** track-before-detect, ground conductivity meters, frequency management, radio interferences, frequency estimation, spectrogram, tracking, VLF

## Abstract

Ground conductivity meters use electromagnetic fields for the mapping of geological variations, like the determination of water amount, depending on ground layers, which is important for the state analysis of embankments. The VLF band is contaminated by numerous natural and artificial electromagnetic interference signals. Prior to the determination of ground conductivity, the meter’s working frequency is not possible, due to the variable frequency of the interferences. Frequency management based on the analysis of the selected band using track-before-detect (TBD) algorithms, which allows dynamical frequency changes of the conductivity of the meter transmitting part, is proposed in the paper. Naive maximum value search, spatio-temporal TBD (ST-TBD), Viterbi TBD and a new algorithm that uses combined ST-TBD and Viterbi TBD are compared. Monte Carlo tests are provided for the numerical analysis of the properties for a single interference signal in the considered band, and a new approach based on combined ST-TBD and Viterbi algorithms shows the best performance. The considered algorithms process spectrogram data for the selected band, so DFT (Discrete Fourier Transform) could be applied for the computation of the spectrogram. Real–time properties, related to the latency, are discussed also, and it is shown that TBD algorithms are feasible for real applications.

## 1. Introduction

Ground conductivity meters allow mapping of geological variations, related to the subsurface using the electromagnetic inductive technique without electrodes or ground contact [1]. Such a technique allows large area surveys with high spatial resolution. Ground conductivity meters are used near to the ground level or far above the ground level (tens of meters) in airborne surveys using a plane or a helicopter. This technique has been used over the last few decades, but alternative electromagnetic measurement techniques are also available, like VLF (very low frequency) and TEM (transient electromagnetic method, alternately called time-domain EM (TDEM) or pulse EM (PEM)) [2]. Different ground properties are examined during such surveys, and one of the most important for Europe is the estimation of the water amount in embankment layers. Broken embankments during winter or spring seasons are the source of local flooding for lowlands, which is dangerous for people, animals and infrastructure, so the protection against flooding is the main motivation for the authors in this project. The rapid control of embankment quality is necessary using airborne techniques mainly, because embankments are long, and direct access to them is limited, depending on weather conditions.

### 1.1. Ground Conductivity Measurements

There is no single technique for ground conductivity measurement, as well as for the estimation of the water profile dependent on depth for embankments. Different operating modes are available in commercial conductivity meters: single frequency, multiple frequency (signals are generated at the same time or time domain multiplexed) and arbitrary waveforms [2]. A sine signal or signals are used with the frequency or frequencies predefined by the manufacturer or selected arbitrarily by the operator. The question about the best technique is still open. The single-frequency or time domain multiplexed frequency modes are assumed in this paper.

**Figure 1:**
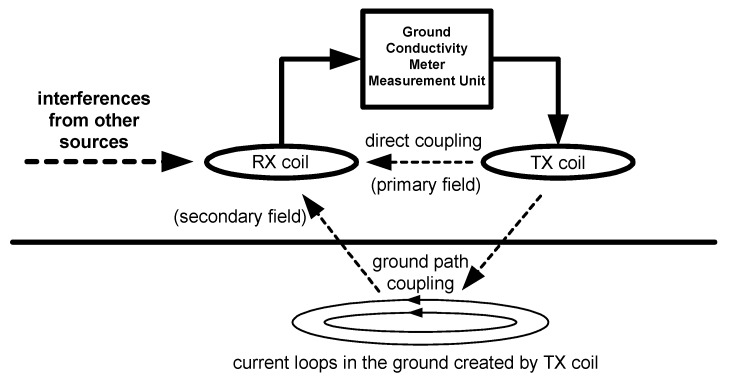
Simplified model of measurements using the ground conductivity meter.

Ground conductivity (quad-phase) and magnetic susceptibility (in-phase) measurements are possible using a two-coil system [3], and the maximum coupling configuration is achieved if two coils share a common plane [1], but minimal coupling configurations (orthogonal) are also possible. Two orientations of the coils are used typically: parallel to the ground (horizontally-oriented coils) and orthogonal to the ground (vertically-oriented coils), so different deep related measurements could be obtained [4,5]. The receiving coil measures two signals together: the primary signal of the transmitting coil (TX) (high power and related to the direct coupling of coils) and the signal related to the ground (very weak and related to the coupling from the transmitting coil to the ground and from the ground to the receiving coil (RX)), which is shown in Figure 1. Two components, quad-phase and in-phase, could be calculated using signals from transmitting and receiving coils [1].

**Figure 2:**
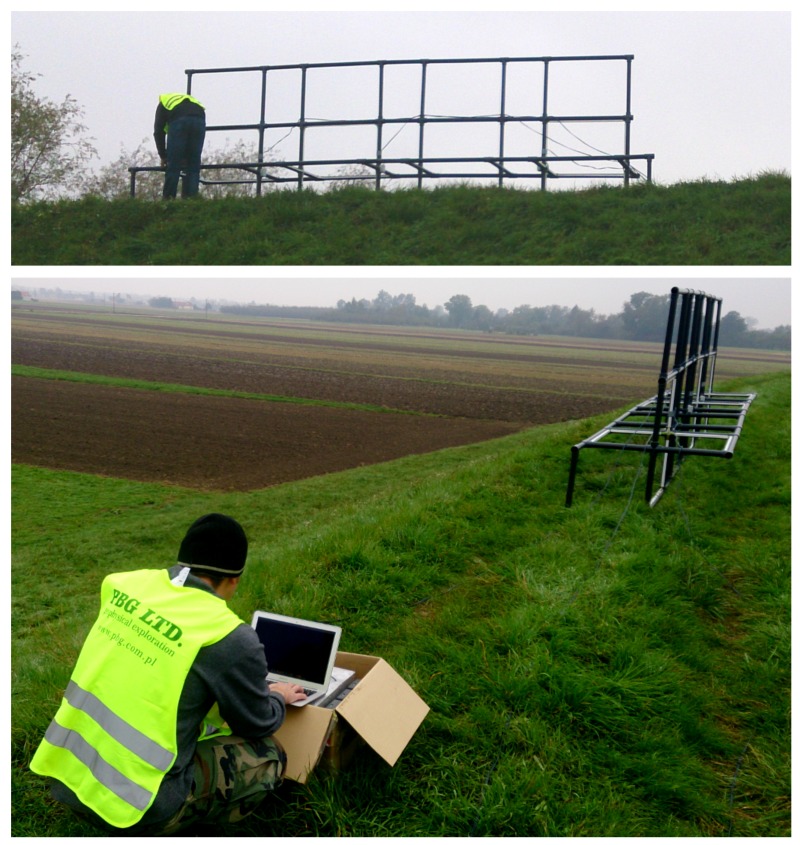
Developed conductivity meter with reconfigurable antenna (four modules: transmitting, spacer, two receiving modules) during ground tests of embankments by PBG Ltd. company (used by permission of PBG: Przedsiȩbiorstwo Badań Geofizycznych sp. z o.o.).

The system developed in the project uses two horizontally- and two vertically-receiving coils (Figure 2) as two modules, but an additional vertical and horizontal coil module could be added. There are two transmitting coils in a single module (vertical and horizontal), so there are eight possible coupling combinations, and two measurements are available for every combination (quad-phase and in-phase). There is an additional module that serves as a spacer for testing different spatial configurations of the antenna. The algorithm for the estimation of the water profile is outside of the paper scope; inversion techniques with a review are considered in, e.g., [6]. The weak signal related to the ground properties could be distorted by different types of external VLF-band signal sources [7].

The detection of weak signals related to the ground is very challenging, and numerous hardware and software techniques have been proposed over the decades. The most typical approach in modern systems is based on the analog-to-digital conversion for all signals and further processing using digital signal processing algorithms, because the stability problems of electronics related to temperature, time and the selection of components values are significantly reduced. Moreover, digital signal processing algorithms allow the detection or suppression of other signal radio interference effects.

**Figure 3:**
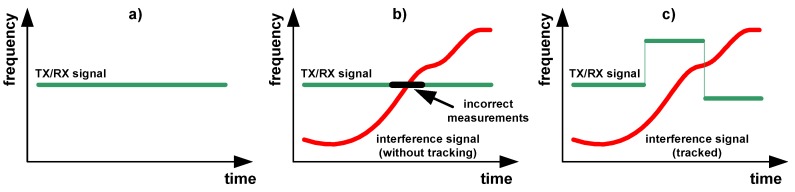
Spectrograms: ground conductivity meter signal for the interference-free case (**a**); incorrect measurements due to interference (**b**); frequency changes of the ground conductivity meter by the application of tracking and prediction for the interference signal (**c**).

The signal is real-time observed in a spectrogram that is necessary for frequency changes of the ground conductivity meter transmitter for omitting the overlap with interference signals [8]; Figure 3. Track-before-detect (TBD) algorithms are proposed in this paper for the frequency estimation of a single radio interference signal (tracked object) in a specified band, because tracking of low SNR interferences should be supported.

### 1.2. Contribution of the Paper and Related Works

There are numerous tracking algorithms for the frequency estimation, but only TBD algorithms support low SNR signal tracking. TBD algorithms could be applied not only for the sine frequency estimation, but also for band tracking, which is important for noise sources [9].

Most important radio interference signals for conductivity meters are considered in Section 2, and some examples are provided. A short introduction to the TBD algorithms is in Section 3, and two of them are emphasized: spatio-temporal TBD (ST-TBD) and Viterbi TBD, in Section 3.2 and Section 3.3, respectively. Both algorithms with their properties are considered comprehensively in [10,11,12,13]. The new approach proposed in this paper assumes combined processing using ST-TBD and Viterbi TBD for quality improvement of frequency tracking. Such combined processing is motivated by previous works [14,15], where two TBD algorithms could give better tracking results and reduce the computation cost. The Monte Carlo test allows the numerical comparison of algorithms and is considered in Section 4. Spectrograms could be generated synthetically for the Monte Carlo test, and an example result for the single tracking case is provided in Section 5.1 for illustrative purposes. Monte Carlo test results are presented in Section 5.2 and show the importance of the proposed tracking solution. A discussion about the obtained results is provided in Section 6.1, and the real-time processing constraints are analyzed in Section 6.2. Final conclusions and further works are formulated in Section 7.

## 2. Selected Examples of Radio Interferences

The possibility of high power transmitter use for improving SNR for a ground-related signal in the case of the occurrence of interference signals is limited by the available power source for mobile devices especially. There are national regulations related to the band access and possible power limitations, as well. A more intelligent approach should be applied, instead of a high power transmitting source. The VLF spectra should be analyzed for the estimation of interference-free narrow bands. The frequency management of the conductivity meter cannot be based on a single-measurement prior survey, so adaptive techniques are necessary, because interference sources are variable.

One of the most important radio interference signals in the VLF band is the power network hum from 50-Hz or 60-Hz generators and power lines. This problem is correlated to the location of embankments. The most important embankments are located near cities or villages, and such areas consist of dense power networks, including high and medium voltage lines.

**Figure 4:**
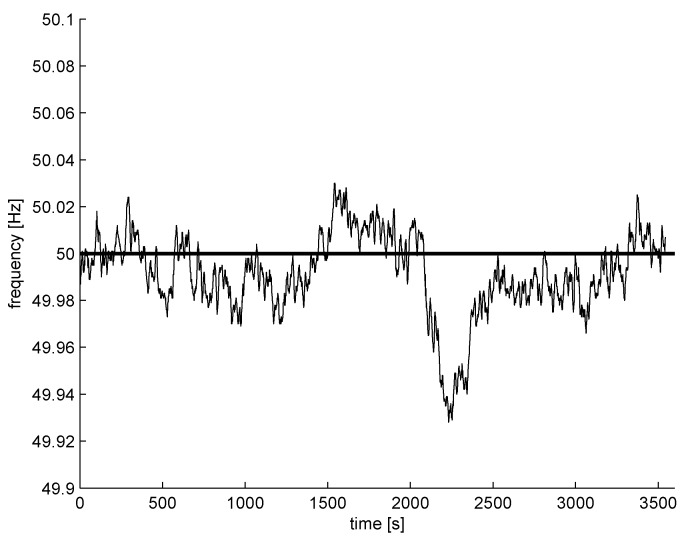
Hourly frequency changes of a power network (50 Hz) example (the data used by permission of Dr. Gobmaier GmbH [17])

The power lines emit a single frequency and multiple harmonics (odds and evens), also. In professional power engineering, only a few first harmonics are considered, but the electromagnetic spectrum is contaminated by hundreds of harmonics. The frequency of the power network is not fixed, and the variability of is controlled [16], so the changes are very small (e.g., 1% of nominal frequency); however, the harmonics’ absolute changes are multiplied by the number of harmonics. Example frequency changes [17] for a single hour are shown in Figure 4 (only 0.14% of nominal frequency). The technique and device for high accuracy metering of power network frequency is considered in [17], for example.

**Figure 5:**
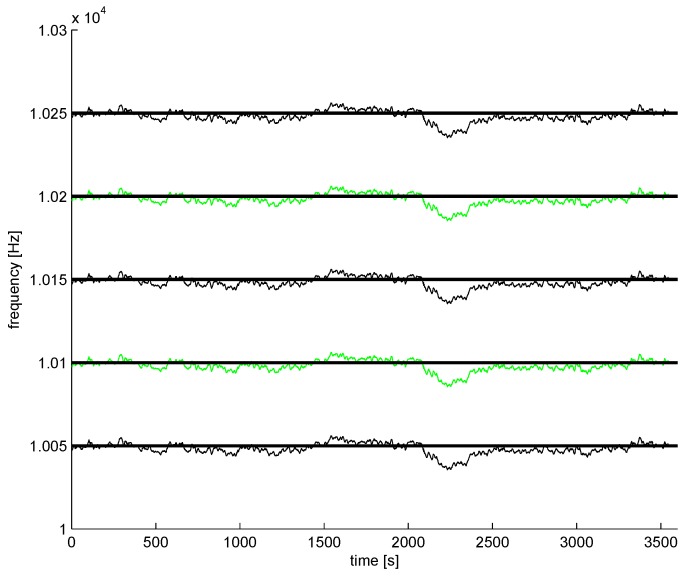
Frequency changes of power network example (Harmonics 201–205): odds are black; evens are green; and ideal frequency lines are black bold lines (calculated for the measurements from Figure 4).

The absolute deviation $\Delta F^{h}$ for considered harmonic *h* could be calculated using the following formulas:

(1) $$F_{ideal}^{h}=h\cdot f_{ideal}$$

(2) $$F_{real}^{h}=h\cdot f_{real}$$

where *F* is the frequency of the *h* harmonic, $f_{ideal}$ is the default power network frequency (50 Hz or 60 Hz) and $f_{real}$ is the momentary frequency. The deviation is obtained from the following formula:

(3) $$\Delta F^{h}=F_{ideal}^{h}-F_{real}^{h}=h\left(f_{ideal}-f_{real}\right)$$

so small changes of the power network frequency are multiplied by the harmonic number *h*. The occupied bandwidth for a longer time period is much wider for higher harmonics, as compared to the first harmonic (50 Hz or 60 Hz) bandwidth occupation. Example bands around 10 kHz (a typical value for commercial conductivity meters [5]) are shown in Figure 5 with odd and even harmonics, so static bands without harmonics are reduced to about 50% of 50 Hz in this case.

Artificial radio interferences are related to the emission by numerous power converters, including fluorescent lamps with electronic ballasts and electronic equipment power supplies [18].

**Figure 6:**
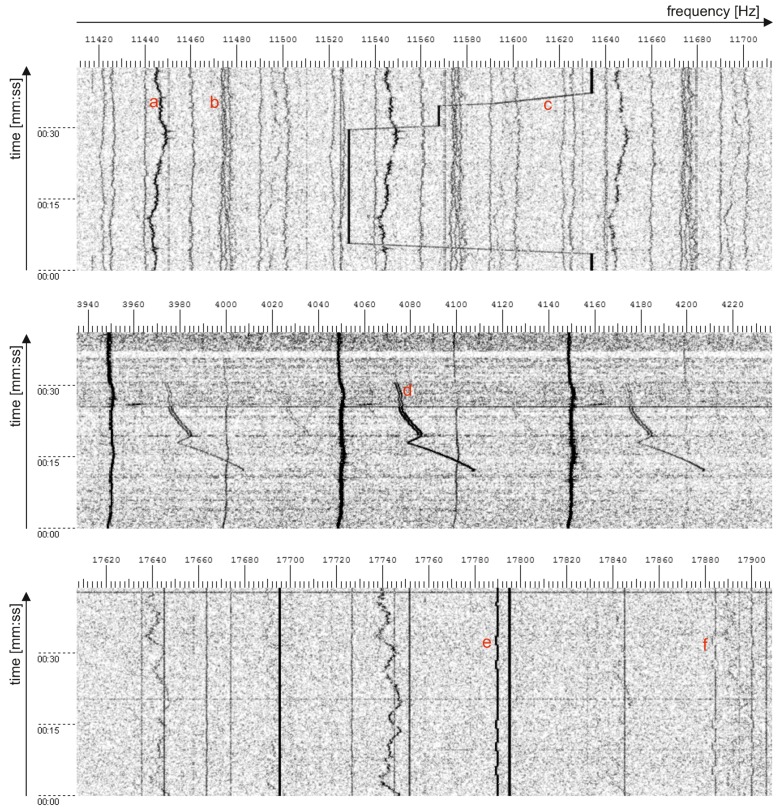
Example artificial radio interference signals: (**a**) power line harmonics; (**b**) unknown sources; (**c**) VLF communication with wide band frequency changes; (**d**) interference from an electric motor; (**e**) VLF communication with narrow band frequency changes; (**f**) modulation artifacts of signal “e”.

Some bands are assigned to radio transmitters, and such bands are well determined and could be omitted. LF radios, navigation systems (e.g., RSDN-20/Alpha (from the Russian: radio-technical long distance navigation system), Loran-C [19]) and time broadcasting systems (e.g., DCF77 [20], WWVB [21] emissions are controlled, and the bands are fixed. Unfortunately, there are many emissions related to military and unknown sources. Some of these emissions are assigned to the single narrow band, and shift keying is used for digital modulation. Another type of emission is based on frequency shifting with large frequency shifts. There are transitions that disturb a band between starting and final frequency during the frequency changes. Natural interferences related to different phenomena are also observed, including pulses and variable frequency signals (whistlers) [22]. The analysis of the spectrum could be applied for the detection of the radio interference signal, so false measurements could be rejected. Simple analog demodulation for a single fixed frequency does not give the opportunity for the detection or the suppression of such artifacts. A few signal types are shown in Figure 6.

## 3. Conventional and Track-Before-Detect Approaches

Tracking is a general task that is considered in numerous signal and image processing applications [23,24,25]. The estimation of frequency (visible as a line in a spectrogram) is very important in speech analysis, vibroacoustics, *etc*. It is assumed that the signal is over the noise background (SNR>1) in most papers. Such a case allows the detection and tracking of spectrogram line changes using simple thresholding techniques, even without tracking, especially for SNR>>1. Advanced line estimation algorithms use a tracking approach based on the detection and tracking. The tracking algorithm improves the detection, if there are numerous false measurements, and the lost observation of the tracked signal occurs. The motion model could be applied, and the tracking algorithm predicts the possible position. The predicted position allows the application of a fixed or adaptive gating technique for the reduction of the search area [23]. Observations are considered that are inside the gate, and the observation with the smallest distance to the center of the gate is a plausible position of the object, for example. Multiple object (multiple spectrogram lines) tracking is possible, but an assignment algorithm is necessary for the track maintenance tasks: creation, removal, merging, splitting and crossing problem solving. The conventional approach uses binary data from the detection part, so the threshold algorithm works as a non-linear filter. Weak signals are rejected using thresholding, so the tracked signal should be over the noise floor.

The signal of the conductivity meter, related to the ground path, is very weak, so the interference signal with the same frequency disturbs the measurements. A single scan of the band of interest prior to the survey is not reliable. The interference signal could be variable (frequency and amplitude), and what is more important, the antenna is directional, so the interference signal could be reduced or amplified depending on the antenna orientation. Maximal coupling with the Earth’s surface-located signal sources could be obtained for vertical antennas especially. Different orientations of antennas are possible during the survey, and some areas of embankments should be checked more carefully, so there is no single geographical orientation of the antenna. Interference signals, which are estimated in real-time, allow the frequency hopping in the transmitting part of the conductivity meter.

The computational cost for tracking systems with the detection and tracking approach is quite low, but the weak signal cannot be estimated. Tracking of interference signals in the selected band using spectrograms by the application of track-before-detect (TBD) algorithms [25] is necessary.

The TBD approach uses the tracking algorithm for raw data, so all signal values are processed and are not omitted by the detection part, like in the conventional detection and tracking approach. After tracking, the detection is applied for the multidimensional state-space. The computational cost of TBD is huge, because all possible trajectories are processed, even if there is no real object in the range, but this tracking algorithm class supports multiple object tracking inherently. The simplest algorithm for TBD uses an accumulative approach, where the signal values are accumulated using the assumed motion model. The state-space could be defined using different techniques, and for a 2D image (spectrogram), the position-velocity (frequency-frequency change) configuration could be used. The spectrogram resolution defines the resolution for the position component of the state-space, and the number of velocities (motion vectors) defines the velocity component of the state-space. The separation of objects depending on motion vectors is obtained. The motion model allows the transition between velocity subspaces if maneuvers are allowed, which are not defined directly by the velocity subspace model. ST-TBD and Viterbi TBD use the Markov model with a predefined set of velocities, so for spectrograms, there is a predefined set of allowed frequency changes (Δf). A maneuver occurs if:

(4) $$\Delta f_{n}^{g}\neq \Delta f_{n+1}^{g}$$

where *g* denotes the particular velocity (particular frequency change) and *n* is the particular discrete index (time moment).

There are numerous TBD algorithms [26], but only deterministic algorithms should be applied. The non-deterministic approach gives computationally-efficient particle filter TBD algorithms [27,28], but not all trajectories are tested. Numerous parameters of TBD algorithms influence the tracking quality, and the selection of parameters requires intensive numerical testing.

ST-TBD and Viterbi TBD algorithms could be used separately. It is possible to use two TBD algorithms together for improved quality or computational cost reduction [14,15].

### 3.1. Naive Approach: Maximum Value Search

**Figure 7:**
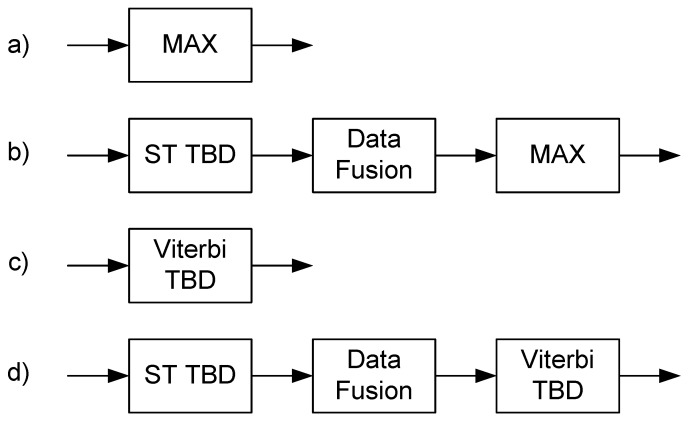
Considered algorithms: (**a**) naive search of maximum value (MAX); (**b**) spatio-temporal track-before-detect (ST-TBD) with data fusion of the state-space and maximum value search; (**c**) Viterbi TBD; (**d**) combined ST-TBD and Viterbi TBD.

The naive approach based on maximum value search (Figure 7a) in current measurement is trivial:

(5) $${\hat{f}}_{n}=\arg\max_{f}X\left(f,n\right)$$

where X(f,n) denotes the cell of the spectrogram (pixel), *n* is the time moment and *f* is the frequency. There is no tracking in this algorithm, and it work well only for very high SNR cases.

### 3.2. Spatio-Temporal Track-Before-Detect Algorithm

The ST-TBD algorithm is a kind of multidimensional recursive filter that uses the Markov model for prediction. This algorithm (Figure 7b) is similar to the simplified likelihood ratio tracker [29] and is a generalization of velocity TBD filters and 3D matched filters [30]. The description of the algorithm is provided in [10,11].

There are two processes inside ST-TBD related to the state-space. The sharpening process is defined by the information update formula, and the blurring process is defined by the motion update formula (prediction). The balance between both processes is controlled by smoothing coefficient *α*. The information update formula is a kind of exponential smoothing filter. High SNR cases require low-valued smoothing coefficients (near zero), and low SNR cases require high-valued (near one) smoothing coefficients. A high-valued smoothing coefficient adds the latency for the detection, during trajectory changes, due to the fixed motion vector set. The Markov matrix defines the transitions between states, but regular matrix calculations are usually not used, because this matrix is sparse, which is very important for time-efficient computations. Values of this matrix are selected to the best fitting for the expected behavior of the tracked object.

The output of the ST-TBD algorithm is the multidimensional state-space, and one of them could be used: from the motion or information update formula [10,11]. The detection could be based on the analysis of the output (state-space) directly or after the data fusion. The detection part assumes a single tracked object for the simplification of the analysis and uses the following formulas:

(6) $${\hat{f}}_{n}=\arg\max_{f}P^{F}\left(n,f\right)$$

(7) $$P^{F}\left(n,f\right)=\max P\left(n,f,.\right)$$

where $P^{F}$ is the data-fused state-space and state-space is defined as P(n,f,g). The spatial component is *f*, and the velocity component is *g*.

### 3.3. Viterbi Track-Before-Detect Algorithm

The Viterbi algorithm is one of the dynamic programming algorithms [31,32] that allows the estimation of the most probable path for certain criteria using multiple observations. This algorithm (Figure 7c) is applied in digital communication for the decoding of convolutional codes [33]. Viterbi TBD could be applied for image processing tasks, e.g., line estimation [34]. Trellis defines transitions between states and could be mapped directly to the image (grid), so states are related to the appropriate pixels. Two-dimensional trellis is directional, and the direction of the trellis is related to the time and orthogonal direction to the frequency. Such a pixel-to-state mapping allows the application of the Markov transition model of the lines directly to the spectrogram image, and the visualization of the process is straightforward.

Viterbi TBD uses the moving window approach for spectrogram analysis. This window spans over all frequency regions (rows of the spectrogram) and a limited number of time moments (columns of the spectrogram). The width of this window is the depth of Viterbi TBD analysis $n_{max}$. The moving window defines the area of analysis, and as the output, the spectrogram pixel position corresponding to the most probable path is obtained. Final and temporal results, for a particular window position, are not used during the computation for other positions of the sliding window.

A detailed description of Viterbi TBD for a single line tracking in provided in [12,15].

The accumulation process in the forward phase of Viterbi TBD is responsible for the TBD behavior. The performance depends on the depth of analysis, so $n_{max}$ cannot be a small value. Larger depths are recommended for a well-fitted transition model to trajectories. Viterbi TBD works very well if maneuvers occur, because Viterbi TBD is not a recursive algorithm in a global sense, where ST-TBD preserves the lags of IIR (Infinite Impulse Response) filters due to recursive processing.

### 3.4. Proposed Approach: Combined ST-TBD and Viterbi TBD

The cascade connection of ST-TBD and the Viterbi TBD algorithm (Figure 7d) is proposed in this paper. The input data for Viterbi TBD is not thresholded using maximum value search, intentionally. This search is applied for the ST-TBD output state-space used separately (Figure 7b), but it is not desired in the combined variant. The ST-TBD output state-space is not binary data, so full available information could be processed by the Viterbi TBD algorithm. The TBD properties of such a combined algorithm depends on the configuration of both algorithms and should be analyzed using Monte Carlo tests.

## 4. Monte Carlo Tests

The analysis of the performance of the algorithm for the spectrogram line tracking is important, but due to the complexity of the tasks, a numerical approach is required. Testing, using real data, is insufficient for the performance analysis and the selection of the best configuration for the tracking algorithms. The Monte Carlo test gives unbiased results, which are required. The computation cost is unfortunately huge, because the parameters of the algorithm and the tested trajectory depend on numerous coefficients. One of the most important advantages of the Monte Carlo test is the possibility of testing a larger space of cases, so even if some cases are not observed in the real data due to the limited size of the database, they could be tested if they are expected by the available knowledge.

Different parameters of the mentioned algorithms are tested using the Monte Carlo approach for synthetically-generated background noise and a single trajectory. Multiple object (multiple frequencies) tracking is possible, but is not considered in this paper.

The Monte Carlo test uses five million cases, because smooth curves in the figures are desired (comb-like shapes in the figures are typical for too small Monte Carlo tests). Two main series of tests are shown, which are marked as “fixed” and “free”, because the Markov model supports a few motion vectors. The “fixed” series occurs if the object motion (frequency changes) is exactly modeled by the motion vectors. This is not a realistic case, usually, but allows testing of the algorithms for a fitted case. The “free” series are for object motions (frequency changes) approximated by the model, which is a realistic case.

There are numerous models of atmospheric noises available for the VLF band especially, which are considered in [35]. A simple Rayleigh distribution is assumed in this paper, because advanced models depend on numerous factors, like latitude and time (month and hour), and the calculation of Rayleigh noise is faster.

**Figure 8:**
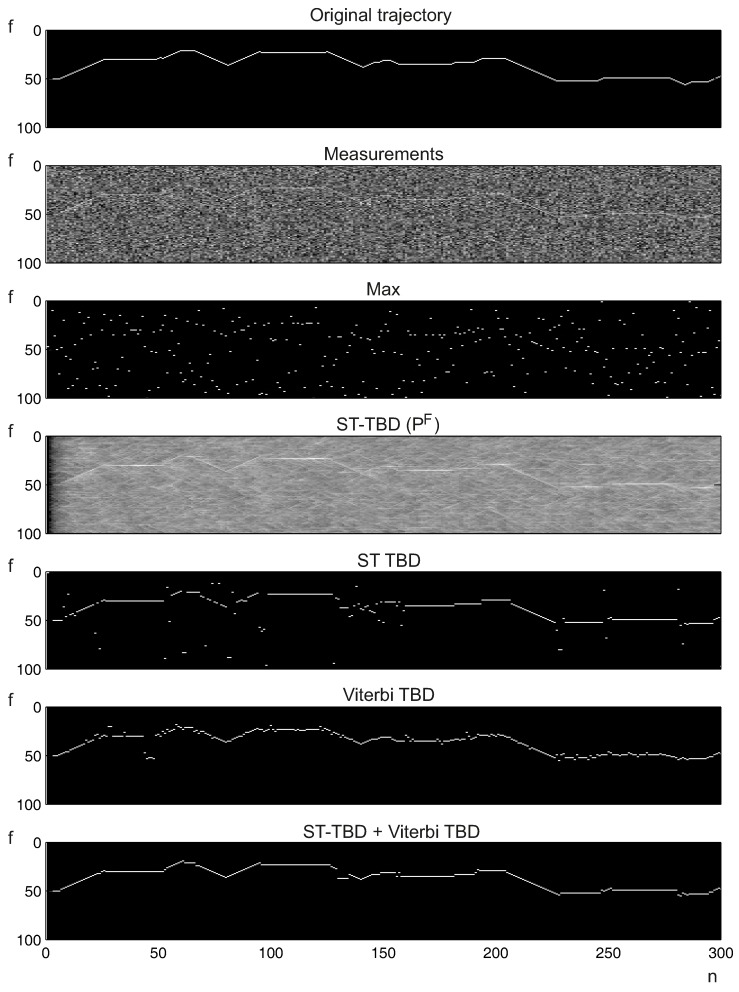
Single case tracking example.

The signal value (tracked interference) is fixed, but randomly selected, and is additive to the noise. The trajectory is synthesized using random lengths with random velocities. The limitation of the trajectory to a region of 100 frequencies is added, and the number of time moments is limited to 300, because longer tests are not necessary; there are many trajectory changes. Moreover, the effects of starting transients in ST-TBD are negligible. The transient time is visible in Figure 8 as a darker part of the state-space after fusion (ST-TBD $P^{F}$). Small values of the smoothing coefficient applied in the test allow for the omitting of transient effects.

The cascade of ST-TBD and Viterbi algorithms (hierarchical TBD system) is an interesting alternative to the separately-used algorithms. The verification of the hypothesis about the possible better performance for such a cascade is considered in the tests. Two algorithms may give a reduction of errors in relation to the other configurations.

A few days of computation are necessary using ten computers (Core 2 Quad, 2.4 GHz) for Monte Carlo tests using MATLAB, when all four processing cores are utilized. The MATLAB code consists of numerous optimizations techniques for the reduction of the processing time.

## 5. Results

### 5.1. Example Tracking Scenario

In this test, the illustrative example tracking is shown, and the signal of the tracked object is still visible to a human (Figure 8). The interference signal is disturbed by atmospheric noise and could be estimated automatically. The maximum value for a specified time moment cannot be used for the determination of the frequency. The considered TBD algorithms allow reliable estimation of the signal frequency.

### 5.2. Monte Carlo Results

The main tests are related to the estimation of the probability of the MAE (mean absolute error) and RMSE (root mean square error) for the difference between the original (known) trajectory and the estimated one. The computation time is the main problem due to the large-scale test and the number of possible analyses, due to the combination of the algorithm parameters.

**Figure 9:**
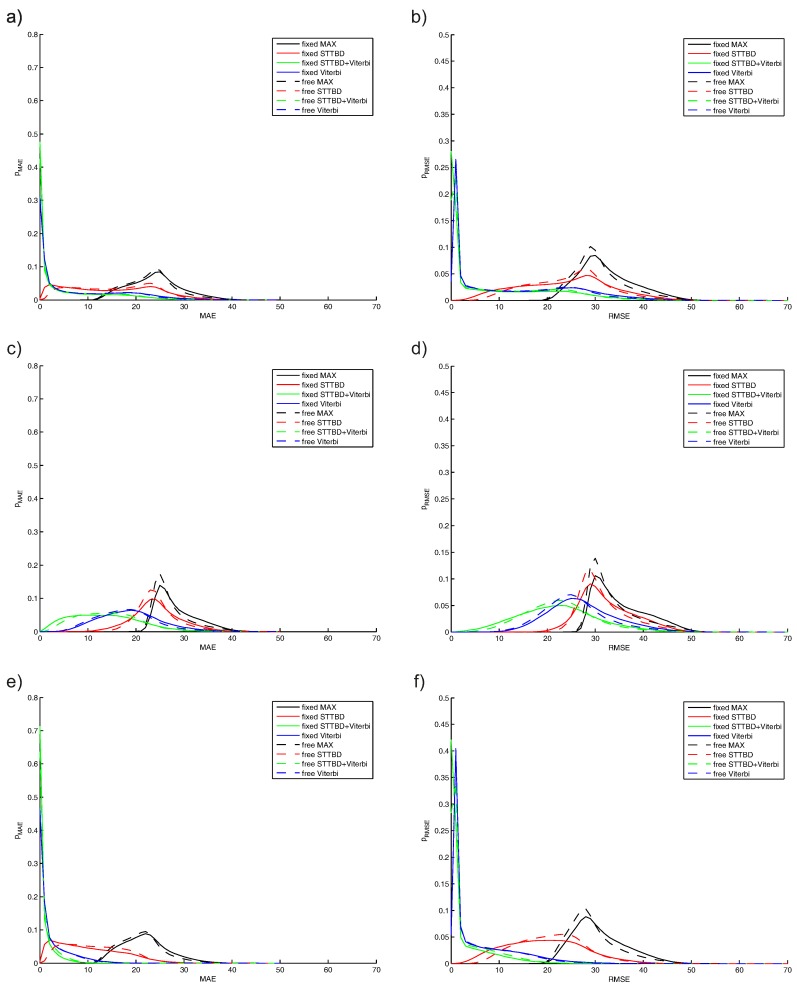
Monte Carlo results: probability of MAE and RMSE: (**a**,**b**) all algorithms for interference amplitude $\left(0–2\right)$; (**c**,**d**) all algorithms for interference amplitude $\left(0–1\right)$; (**e**,**f**) all algorithms for interference amplitude $\left(1–2\right)$).

The first set of results is shown in Figure 9, and all considered algorithms are compared for fixed and free variants independently on the interfering signal amplitude (a,b). Those tests show errors in the estimation of position on the spectrogram without prior knowledge about the interfering signal amplitude. The interference signal amplitude is in the $\left(0–2\right)$ range. The case of SNR<1 (signal amplitude from $\left(0–1\right)$) is presented in Figure 9c,d. The case of SNR>1 (signal amplitude from $\left(1–2\right)$) is shown in Figure 9e,f. The smoothing coefficient is from $\left(0.3–0.8\right)$, and the depth of analysis is from $n_{max}=\langle 10–20\rangle$.

**Figure 10:**
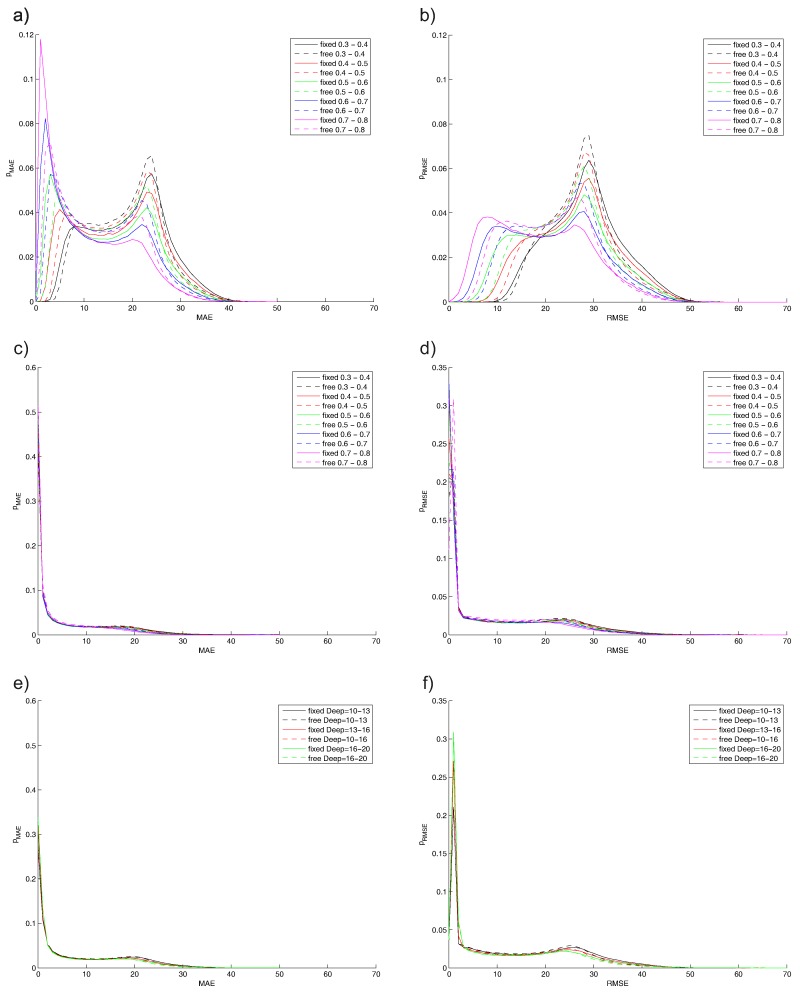
Monte Carlo results: probability of MAE and RMSE (**a**,**b**) ST-TBD depending on the smoothing coefficient; (**c**,**d**) ST-TBD and Viterbi TBD depending on the smoothing coefficient; (**e**,**f**) Viterbi TBD depending on the depth of analysis.

The second set of results is shown in Figure 10 for TBD algorithms only. ST-TBD results, depending on the smoothing coefficient, are shown in Figure 10a,b, where a few ranges of this coefficient are arbitrarily assumed. Similar results are depicted in Figure 10c,d for the combined ST-TBD and Viterbi TBD system. The last results, depicted in Figure 10e,f, are related to the Viterbi TBD and different ranges of depths of analysis.

## 6. Discussion

### 6.1. Algorithms’ Comparison

The naive algorithm using the maximum value search has poor performance, and this is visible in Figure 9a, where the MAE is between 10 and 40. This error probability consists of higher error values (20–40) for SNR<1, which is shown in Figure 9c, and lower error values (10–35) for SNR>1, shown in Figure 9e. The RMSE error for the naive algorithm is similar.

The separation of the total error for another algorithm is possible and desired, because two modes are visible, especially for Viterbi TBD.

The probabilities of the MAE and RMSE error for SNR<1 are reduced if different TBD algorithms are changed. The highest errors are for ST-TBD, lower for Viterbi TBD and minimal for combined ST-TBD and Viterbi TBD (Figure 9c,d) for SNR<1. A very high probability of small errors exists for Viterbi and combined ST-TBD and Viterbi TBD for SNR>1. The performance of ST-TBD is low due to the SNR paradox for recursive algorithms [36] (the high SNR case is preferred for tracking, but degrades tracking if a maneuver occurs, so the low SNR case is better, because smaller error occurs during the maneuver).

The smoothing coefficient is very important in ST-TBD, because this algorithm preserves the behavior of the exponential filter. The selection of this coefficient could be based on the numerical evaluation. MAE and RMSE errors are shown in Figure 10a,b. Higher values of 0.7–0.8 or more should be used for tracking improvement using this algorithm for the assumed model.

A similar conclusion for the combined ST-TBD and Viterbi TBD system could be obtained. Both kinds of errors are shown in Figure 10c,d, but the differences between the probability curves are low, so the sensitivity of the selection of the smoothing coefficient is low, also. These results are important and show the additional correction of the results obtained from ST-TBD by the Viterbi algorithm. The advantages of the proposed combined system, over ST-TBD used separately, are visible if MAE and RMSE are compared, respectively. The maximum value of MAE is reduced, so the worst case is about 10 pixels lower. The high peak around the zero value of MEA and RMSE (probability of about 0.3) means that the correct position is estimated, and the probability of higher errors is reduced.

Increasing of the depth analysis in Viterbi TBD, used separately, reduces the higher errors, which is shown in Figure 10e,f. The differences are not high, for the considered range of $n_{max}$, so the sensitivity for the value selection is rather low.

The comparison of “fixed” and “free” test variants shows the importance of the fitting of the Markov model to the behavior of the tracked object (frequency changes); but the differences are not high, and the shape of the probability curves is preserved.

There are possibly many additional configurations, like Viterbi and ST-TBD together, or two or more combinations of the same, or different TBD algorithms. The filtering of trajectories using linear or nonlinear filters (e.g., median filters), as well as the application of conventional tracking algorithms, like the Kalman filter, are possible, also.

### 6.2. Real-Time Constraints

Real-time processing of the spectrograms is necessary for conductivity meter applications. The ST-TBD algorithm requires many computations, especially for 2D tracking scenarios, but this application is different. The input space is 1D (row/column of spectrogram) for real-time processing, so the computation cost is fortunately low. The number of motion vectors could be small, which was considered in the previous section, and real-time processing is possible using a modern CPU or GPGPU (General-Purpose computing on Graphics Processing Unit). The ST-TBD input data rate *R* depends on the sampling frequency $f_{s}$, and the number of samples *N* processed by DFT is:

(8) $$R=\frac{f_{s}}{N}$$

Typical values of this rate are about one or few input spaces per second, so the main computational cost is related to spectral analysis, not to the ST-TBD processing. The latency of ST-TBD is meaningful in comparison with the spectral analysis ($N/f_{s}$ in seconds).

Viterbi TBD requires more sophisticated computations, because the code is not MAC (Multiply-and-ACcumulate) oriented, like ST-TBD, but modern computation devices are fast enough, so the main computation effort is related to the spectrogram processing (DFT). The latency depends on the depth of analysis (sliding window size). The direct implementation of the Viterbi TBD process assumes the processing of $n_{max}$ columns starting from the oldest to the current during the forward phase, so this approach gives a very large latency:

(9) $$n_{max}\frac{N}{f_{s}}$$

that is not acceptable for real-time processing (e.g., tens of seconds). The solution is possible by the reversion of time direction for the current position of the sliding window inside this window. Viterbi TBD does not use previous results, which are obtained for different sliding window positions, so the time direction could be reversed. Starting measurements should be used as the current measurements, and the forward phase should be computed using previous measurements. Such a time reversal gives a smaller latency, like in ST-TBD:

(10) $$\frac{f_{s}}{N}$$

The combined ST-TBD and Viterbi system preserves the limitation of both algorithms, so the latency is low, similarly.

## 7. Conclusions

Tracking of the radio interference signal reduces the possibility of weak signal distortion related to the ground, which is important for conductivity metering. Three proposed TBD systems are compared using Monte Carlo tests and the proposed combined ST-TBD, and the Viterbi TBD system gives the best quality of tracking. The computational cost is low for such tracking algorithms, and the latency could be reduced to the minimal possible delay.

Monte Carlo tests are used for the validation of the behavior for single radio interference, and further works will be related to the extension for multiple interference signals. ST-TBD is a multi-object tracking algorithm, so Viterbi TBD should be extended for multiple object cases, and an example approach is considered in [37].

The estimation of frequencies, related to interferences, allows the control of the conductivity meter transmitting part. Moreover, the prediction from the TBD algorithm or an additional predictor could be applied for the estimation of the possible state of interferences.
